# I Hear You, I Feel You: Evaluating the Impact of Hearing Loss among Rural Homebound Older Veterans

**DOI:** 10.1093/geroni/igaf122.3606

**Published:** 2025-12-31

**Authors:** Nathan Sheets, Elizabeth Reilly, Sandra Sanchez-Reilly, Jose Mendoza, Michael Mader

**Affiliations:** University of Texas Health Science Center at San An, San Antonio, Texas, United States; South Texas Veterans Health Care System, San Antonio, Texas, United States; South Texas Veterans Health Care System and the University of Texas Health Science Center at San Antonio, San Antonio, Texas, United States; South Texas Veterans Health Care System, San Antonio, Texas, United States; South Texas Veterans Health Care System, San Antonio, Texas, United States

## Abstract

**Background:**

Over 50% older Veterans (OV) live with disabling hearing loss (HL), often undiagnosed and/or untreated. HL consequences include increased depression/dementia risk, PTSD exacerbation, social isolation, caregiver-burden and adverse events when hospitalized. Homebound rural OV consequences of HL remain unknown.

**Objective:**

Investigate the relationship between HL, quality-of-life and caregiver-burden among homebound rural OV

**Methods:**

Homebound rural OV (Del Rio, TX) were evaluated for HL (audiometer), caregiver-burden (Zarit), hearing-loss-impact-on-quality-of-life measured with Hearing-Handicap-Scale (HHS), and overall quality-of-life (SF-12).

**Results:**

N-19. Median Age 77 (74-81), 58% Hispanic, 100% male. Spouses were main caregivers. 58% had three-­or-more chronic conditions: 63% mental-health-related, 26% cardiac, 21% pulmonary. 42% reported toxic exposures. 90% OV had HL; 69% found to have moderate-to-severe HL (41-70 dB of loss, 84% bilateral). 57% OV exposed to agent-orange had severe HL (p = 0.089). Caregiver-burden scores were higher among OV with severe HL (p=NS) and quality-of-life was not significantly correlated with HL (Physical: p = 0.93, Mental: p = 0.47, Total: p = 0.71). OV with Mild or Normal HL had lower total values of HHS vs. moderate HL (p = 0.035) and severe HL (p = 0.016). Further, both emotional and social HHS components showed negative significant correlations with severe HL (p = 0.013 and p = 0.021, respectively).

**Conclusions:**

Homebound OV likely suffer from multiple chronic conditions including HL (90%). Severe HL affects quality-of-life and causes caregiver-burden. Self-perceived HL social/emotional impairment significantly correlates to HL severity. In-home interventions such as standardized screening and provision of simpler/cost-effective devices (pocket-talker, alternative to hearing-aids) are needed to avoid further decline among homebound rural populations.

